# Pre-diagnosis tea and coffee consumption and survival after a diagnosis of ovarian cancer: results from the Ovarian Cancer Association Consortium

**DOI:** 10.1038/s41416-024-02792-7

**Published:** 2024-07-18

**Authors:** Christina M. Nagle, Torukiri I. Ibiebele, Elisa V. Bandera, Daniel Cramer, Jennifer A. Doherty, Graham G. Giles, Marc T. Goodman, Gillian E. Hanley, Holly R. Harris, Allan Jensen, Susanne K. Kjaer, Alice W. Lee, Roger L. Milne, Bo Qin, Jean Richardson, Naoko Sasamoto, Weiva Sieh, Kathryn L. Terry, Linda Titus, Britton Trabert, Nicolas Wentzensen, Anna H. Wu, Andrew Berchuck, Malcolm Pike, Celeste Leigh Pearce, Penelope M. Webb

**Affiliations:** 1 Gynaecological Cancers Group, Population Health Program, QIMR Berghofer Medical Research Institute, Brisbane, QLD Australia; 2 Cancer Epidemiology and Health Outcomes, Rutgers Cancer Institute, New Brunswick, NJ USA; 3 Obstetrics and Gynecology Epidemiology Center, Department of Obstetrics and Gynecology, Brigham and Women’s Hospital and Harvard Medical School, Boston, MA USA; 4 Harvard T.H. Chan School of Public Health, Boston, MA USA; 5 Huntsman Cancer Institute, Department of Population Health Sciences, University of Utah, Salt Lake City, UT USA; 6 Cancer Epidemiology Division, Cancer Council Victoria, Melbourne, VIC Australia; 7 Centre for Epidemiology and Biostatistics, Melbourne School of Population and Global Health, The University of Melbourne, Melbourne, VIC Australia; 8 Precision Medicine, School of Clinical Sciences at Monash Health, Monash University, Clayton, VIC Australia; 9 Cancer Prevention and Control Program, Samuel Oschin Comprehensive Cancer Institute, Cedars-Sinai Medical Center, Los Angeles, CA USA; 10 Community and Population Health Research Institute, Department of Biomedical Sciences, Cedars-Sinai Medical Center, Los Angeles, CA USA; 11 University of British Columbia, Department of Obstetrics & Gynaecology, Vancouver, BC Canada; 12 Program in Epidemiology, Division of Public Health Sciences, Fred Hutchinson Cancer Center, Seattle, WA USA; 13 Department of Epidemiology, School of Public Health, University of Washington, Seattle, WA USA; 14 Department of Virus, Lifestyle and Genes, Danish Cancer Institute, Copenhagen, Denmark; 15 Department of Gynecology, Rigshospitalet, University of Copenhagen, Copenhagen, Denmark; 16 Department of Public Health, California State University, Fullerton, Fullerton, CA USA; 17 Department of Population and Public Health Sciences, Keck School of Medicine, University of Southern California, Los Angeles, CA USA; 18 Department of Epidemiology, University of Texas MD Anderson Cancer Center, Houston, TX USA; 19 Department of Epidemiology, Geisel School of Medicine at Dartmouth, Hanover, NH USA; 20 Metabolic Epidemiology Branch, Division of Cancer Epidemiology and Genetics, National Cancer Institute, Bethesda, MD USA; 21 Department of Obstetrics and Gynecology, University of Utah, Huntsman Cancer Institute, Salt Lake City, UT USA; 22 Clinical Genetics Branch, Division of Cancer Epidemiology and Genetics, National Cancer Institute, Bethesda, MD USA; 23 Division of Gynecologic Oncology, Duke University School of Medicine, Durham, NC USA; 24 Department of Epidemiology and Biostatistics, Memorial Sloan Kettering Cancer Center, New York, NY USA; 25 Department of Epidemiology, University of Michigan School of Public Health, Ann Arbor, MI USA; 26 University of Queensland, School of Public Health, Brisbane, QLD Australia

**Keywords:** Ovarian cancer, Epidemiology

## Abstract

**Background:**

Tea and coffee are the most frequently consumed beverages in the world. Green tea in particular contains compounds with potential anti-cancer effects, but its association with survival after ovarian cancer is uncertain.

**Methods:**

We investigated the associations between tea and coffee consumption before diagnosis and survival using data from 10 studies in the Ovarian Cancer Association Consortium. Data on tea (green, black, herbal), coffee and caffeine intake were available for up to 5724 women. We used Cox proportional hazards regression to estimate adjusted hazard ratios (aHR) and 95% confidence intervals (CI).

**Results:**

Compared with women who did not drink any green tea, consumption of one or more cups/day was associated with better overall survival (aHR = 0.84, 95% CI 0.71–1.00, *p*-trend = 0.04). A similar association was seen for ovarian cancer-specific survival in five studies with this information (aHR = 0.81, 0.66–0.99, *p*-trend = 0.045). There was no consistent variation between subgroups defined by clinical or lifestyle characteristics and adjustment for other aspects of lifestyle did not appreciably alter the estimates. We found no evidence of an association between coffee, black or herbal tea, or caffeine intake and survival.

**Conclusion:**

The observed association with green tea consumption before diagnosis raises the possibility that consumption after diagnosis might improve patient outcomes.

## Background

Ovarian cancer is the seventh most common cause of cancer death among women worldwide and although 5-year survival has improved over time, it remains below 50% [1, 2]. Tea and coffee, two of the most commonly consumed beverages worldwide, contain compounds that have the potential to influence ovarian cancer risk and survival. Green tea, in particular, has an abundance of bioactive polyphenols including catechins. Epigallocatechin‐3‐gallate (EGCG) is the most biologically active catechin in green tea and in vitro studies in human cancer cell lines have suggested that in addition to functioning as an antioxidant, it may also inhibit angiogenesis and stimulate apoptosis by negatively regulating the cell cycle (reviewed in [3, 4]).

Meta-analyses suggest a possible inverse relationship between tea consumption, particularly green tea, and risk of ovarian cancer (including 5 case-control studies with 2994 cases for green tea) [5], but no strong association with coffee or caffeine consumption (15 cohort studies; 3927 cases) [6]. Fewer studies have explored the relationships between tea and coffee consumption and survival following diagnosis with ovarian cancer but the limited data indicate a possible benefit with greater green tea intake [7–9]. We used data from Ovarian Cancer Association Consortium to assess the associations between the consumption of these common beverages and survival following a diagnosis of ovarian cancer. Our primary hypothesis was that consumption of green tea, but not other types of tea or coffee/caffeine, would be associated with better survival.

## Methods

We included primary data from one cohort, one case-only and eight case-control studies of ovarian cancer (Table 1) participating in the Ovarian Cancer Association Consortium (OCAC) that also provided dietary data through the Multidisciplinary Ovarian Cancer Outcomes Group (MOCOG). Five studies were from the USA (Diseases of the Ovary and their Evaluation Study [DOV] [10], Hawaii Ovarian Cancer Study [HAW] [11], Los Angeles County Case Control Studies of Ovarian Cancer [LAC] [12], New England Case–Control Study of Ovarian Cancer [NEC] [13], New Jersey Ovarian Cancer Study [NJO] [14]), two from Europe (Danish Malignant Ovarian Tumour Study [MAL] [15], Polish Ovarian Cancer Study [POL] [16]), and three from Australia (Australian Ovarian Cancer Study [AUS] [17], Melbourne Collaborative Cohort Study [MCC] [18] and Ovarian Cancer Prognosis and Lifestyle Study [OPL] [19]). Women missing dietary (*N* = 2322) or follow-up information (*N* = 307), with more than 2 years between diagnosis and interview (*N* = 169) or implausible energy intake ( > 3 SD from the study mean, *N* = 58) were excluded. In primary analyses, we also excluded 240 women who died in the first year following diagnosis leaving an analysis cohort of 5724 women.

**Table 1:** Characteristics of the contributing studies.

Study acronym	Location and Type of study	Year of diagnosis	Number of cases (*N* = 5964)^a^	Deaths (%)	5-year mortality (%)	Median Follow-up in years (range)^b^	Data available^c^
AUS	Australia Case-control	2002–2005	1159	819 (71%)	605 (52%)	10.8 (9.1–13.5)	B/C/D/G/H
DOV	USA Case-control	2002–2009	1040	624 (60%)	440 (43%)	11.7 (7.5–16.1)	B/C
HAW	USA Case-control	1994–2008	378	218 (58%)	149 (40%)	10.2 (4.0–21.0)	B/C/D/G/H
LAC	USA Case-control	1994–2004	639	439 (69%)	281 (44%)	15.9 (4.1–26.2)	B/C/D/G
MAL	Europe Case-control	1994–1998	93	74 (80%)	50 (55%)	22.3 (20.2–23.4)	B/C
MCC	Australia Cohort	1990–2008	99	74 (75%)	56 (58%)	19.5 (11.0–25.7)	B/C/H
NEC	USA Case-control	1992–2008	1386	788 (57%)	536 (39%)	12.9 (6.9–22.8)	B/C/D
NJO	USA Case-control	2005–2008	196	116 (59%)	73 (38%)	9.0 (5.9–11.2)	B/C/G
OPL	Australia Case-only	2012–2015	718	373 (52%)	316 (44%)	6.6 (0.8–8.7)	B/C/D/G/H
POL	Europe Case-control	2000–2003	256	140 (55%)	129 (53%)	5.3 (0.1–7.2)	B/C

*AUS* Australian Ovarian Cancer Study, *DOV* Diseases of the Ovary and their Evaluation Study, *HAW* Hawaii Ovarian Cancer Study, *LAC* Los Angeles County Case–Control Studies of Ovarian Cancer, *MAL* Danish Malignant Ovarian Tumour Study, *MCC* Melbourne Collaborative Cohort Study, *NEC* New England Case–Control Study of Ovarian Cancer, *NJO* New Jersey Ovarian Cancer Study, *OPL* Ovarian Cancer Prognosis and Lifestyle Study, *POL* Polish Ovarian Cancer Study.

^a^Women who completed the diet questionnaire > 2 years after diagnosis are excluded; deaths within the first year are included.

^b^Among women who had not died.

^c^*B* = Black tea, *C* = Coffee – all, *D* = Decaffeinated vs. caffeinated coffee, *G* = Green tea, *H* = Herbal tea; All except MAL and POL also had total caffeine intake.

Researchers at QIMR Berghofer Medical Research Institute were responsible for pooling and harmonising the dietary data. All studies were approved by their relevant institutional review board and all participants provided informed consent.

### Tea, coffee and caffeine intake

Tea and coffee consumption prior to diagnosis was assessed using food frequency questionnaires (FFQ) that asked about the year prior to diagnosis (or 5 years in POL). All studies provided information about total coffee and black tea consumption, five also asked about green tea (AUS, HAW, LAC, NJO, OPL), four about herbal teas (AUS, HAW, MCC, OPL) and five asked separately about caffeinated and decaffeinated coffee (AUS, HAW, LAC, NJO, OPL). All studies except MAL and POL provided information about total caffeine intake. Coffee and tea consumption was categorised as 0, <1, 1–2.49 and ≥ 2.5 cups/day; the top two groups were combined for decaffeinated coffee, green and herbal tea as few women drank more than 2.5 cups per day. Study-specific quartiles were created for caffeine intake.

### Covariate information

Information regarding factors potentially associated with diet or survival was accessed from the central harmonised OCAC database. This included age at diagnosis (years); race/ethnicity (categorised as non-Hispanic white, Hispanic, Asian, Black; racial groups with ≤ 15 women in a study were combined as ‘other’); education (less than high school, completed high school, some post-high school education); body mass index (BMI) ( < 25, 25–29, ≥ 30 kg/m^2^) reported for the period one (AUS, LAC, NEC, NJO) or five years prior to diagnosis (DOV, HAW, MAL, OPL, POL) or at cohort entry (MCC); pre-diagnosis smoking status (never, former, current); pre-diagnosis physical activity (‘active’, ‘inactive’ [20]); and pre-diagnosis menopausal hormone therapy (MHT) use (any, none). Clinical information included tumour stage (local, regional, distant), histotype (high grade serous, low grade serous, mucinous, endometrioid, clear cell, other) and amount of residual disease remaining after surgery (none, any; AUS, HAW, MAL, NEC, OPL only). Missing indicators were assigned to non-dietary variables that were completely or partially missing for a study (see Table 1 for numbers missing).

### Clinical and survival data

Each study reported vital status and survival time, calculated from date of diagnosis to date of death from any cause or date of last follow-up for those still alive. Cause of death information was available for five studies (AUS, DOV, HAW, MAL, OPL) but, in these studies, the vast majority of deaths were from ovarian cancer (92% overall, 95% in the first five years).

### Statistical analyses

Data from the studies were pooled. We combined women missing stage information (3%) with advanced cancers, and combined the small group of low-grade serous cancers (*N* = 179) with mucinous cancers, as in both cases the groups had very similar adjusted survival outcomes. Because the vast majority of deaths were from ovarian cancer, we used death from any cause as the primary outcome to maximise power and conducted a secondary analysis looking at death from ovarian cancer in the subset of studies with this information. In our primary analyses, we excluded women who died in the first year; we therefore left-truncated survival to one year after diagnosis or, in case-control studies, the date of questionnaire completion if this occurred more than one year after diagnosis. This was to avoid immortal time bias and reduce the potential of survivorship bias arising from the exclusion of eligible women who died before recruitment.

We used Cox proportional hazards regression to estimate hazard ratios (HR) and 95% confidence intervals (CI) for the associations with survival. All models were adjusted for age (in years) and stratified by study, tumour stage and histotype as the baseline hazard varied greatly by these factors. Final models were also adjusted for race, education, BMI, smoking status and physical activity; additional adjustment for total energy intake, intake of dairy foods or sugar (which might preferentially be added to some types of hot beverage), year of diagnosis, interval between diagnosis and recruitment, and MHT use did not alter the estimates so these variables were not included. We assessed linear trends by assigning each group a value from 0 (lowest) to 2 or 3 (highest). The only variables to violate the proportional hazards assumption were age at diagnosis and stage; log-time interactions were not included in the final models as their inclusion did not alter the estimates of interest.

Finally, we also investigated whether associations differed according to study, stage of disease (local/regional vs. distant), histotype (high-grade serous cancer [HGSC] vs. other), race, residual disease, menopausal status, BMI, smoking or physical inactivity. Analyses were performed using SAS version 9.4 (SAS Institute, Cary, NC, USA) and STATA version 13 (College Station, TX, USA). Code can be accessed from the investigators if required.

## Results

Table 2 shows the characteristics of the combined study sample. The average age at diagnosis was 57 years and most women were diagnosed with advanced HGSC. As expected, older age, more advanced disease at diagnosis, HGSC, presence of residual disease after surgery, smoking and obesity (BMI > 30 kg/m^2^) were associated with worse survival.

**Table 2:** Characteristics of the study population and associations with overall survival.

Characteristics at diagnosis	Subgroup	*N*	%	Age- and stage-adjusted HR (95% CI)
Age (mean & range)		5724	57.1 (20–87)	
Vital status	Alive	2295	40.1	
	Deceased	3429	59.9	
Year of diagnosis	1992-9	974	17.0	1.00
	2000-3	1109	19.4	0.77 (0.67–0.89)
	2003-6	2083	36.4	0.76 (0.66–0.87)
	2006-9	761	13.3	0.75 (0.64–0.88)
	2012-5	797	13.9	0.68 (0.50–0.93)
Age group	< 40	346	6.0	0.58 (0.48–0.70)
	40–49	1058	18.5	0.79 (0.71–0.88)
	50–59	1858	32.5	1.00 (Ref)
	60–69	1676	29.3	1.15 (1.06–1.25)
	70+	786	13.7	1.42 (1.28–1.57)
Tumour stage	Local	1122	19.6	1.00
	Regional	939	16.4	1.64 (1.39–1.94)
	Distant	3488	60.9	6.05 (5.31–6.90)
	Missing	175	3.1	
Histotype	High grade serous	3436	60.0	1.00
	Low grade serous	179	3.1	0.66 (0.54–0.80)
	Mucinous	313	5.5	0.61 (0.49–0.76)
	Endometrioid	844	14.7	0.54 (0.47–0.62)
	Clear cell	377	6.6	0.74 (0.62–0.89)
	Mixed/Other	575	10.0	0.88 (0.78–0.99)
Residual disease after surgery^a^	Nil	1088	19.0	1.00
	Any	878	15.3	1.94 (1.70–2.21)
	Missing	3758	65.7	
Menopausal status^b^	Pre- and perimenopause	1543	27.0	1.00
	Post menopause	4045	70.7	1.09 (0.97–1.23)
	Missing	136	2.4	
Menopausal hormone	No	3760	65.7	1.00
use	Yes	1856	32.4	0.92 (0.85–0.99)
	Missing	108	1.9	
Race/Ethnicity	Non-Hispanic White	4852	84.8	1.00
	Hispanic	141	2.5	0.92 (0.72–1.18)
	Black	64	1.1	1.11 (0.76–1.63)
	Asian	393	6.9	0.97 (0.83–1.14)
	Other	274	4.8	1.06 (0.90–1.26)
Education	Less than high school	1055	18.4	1.00
	High school	2836	49.6	1.01 (0.92–1.11)
	College or University	1784	31.2	0.94 (0.85–1.05)
	Missing	49	0.9	
Smoking status	Never smoker	3097	54.1	1.00
	Former smoker	1842	32.2	1.13 (1.05–1.22)
	Current smoker	743	13.0	1.25 (1.13–1.39)
	Missing	42	0.7	
Body Mass Index	<25.0	2762	48.3	1.00
(kg/m^2^)	≥ 25 to < 30	1668	29.1	0.93 (0.86–1.01)
	≥ 30	1243	21.7	1.12 (1.03–1.22)
	Missing	51	0.9	
Physical activity^c^	Inactive	1255	21.9	1.00
	Active	3986	69.6	0.93 (0.85–1.01)
	Missing	483	8.4	

*CI* confidence interval, *HR* hazard ratio, adjusted for age and stratified by site.

^a^Residual disease information was not available for DOV, LAC, MCC, NJO or POL.

^b^Menopausal status data were not available for MCC.

^c^Physical inactivity data were not available for MCC or POL.

We found no evidence of an association between consumption of coffee (any, caffeinated or decaffeinated), black tea, herbal tea or caffeine before diagnosis and overall survival (Table 3). Consumption of one or more cups of green tea per day was, however, associated with significantly better survival in both the minimal and fully-adjusted models (fully-adjusted HR 0.84, 95% CI 0.71–1.00, *p*-trend = 0.04). There was no evidence of heterogeneity among the five studies that contributed to the green tea analysis (AUS, HAW, LAC, NJO, OPL; I^2^ = 0.17, *p* = 0.3) and the effect estimate was below 1.0 in four of the studies, with a non-significant positive association in LAC (Supplementary Figure). When we omitted studies individually the pooled estimates ranged from 0.79 (0.66–0.95) omitting LAC to 0.87 (0.70–1.07) omitting OPL.

**Table 3:** Associations between coffee and tea consumption and overall survival after a diagnosis of ovarian cancer.

			None	<1 cup/day	1–2.49 cups/day	≥ 2.5 cups/day	
	Model^a^	N^b^	Reference	HR (95% CI)	HR (95% CI)	HR (95% CI)	*P*-trend^c^
Coffee	1	5713	1.0	0.93 (0.83–1.04)	0.97 (0.88–1.08)	0.99 (0.89–1.10)	0.8
	2	5688	1.0	0.93 (0.83–1.04)	0.97 (0.88–1.07)	0.97 (0.87–1.08)	0.8
Caffeinated	1	4108	1.0	0.93 (0.83–1.05)	0.94 (0.84–1.05)	1.00 (0.89–1.12)	0.97
	2	4096	1.0	0.94 (0.83–1.05)	0.93 (0.83–1.05)	0.99 (0.88–1.10)	0.8
Decaffeinated^d^	1	4108	1.0	1.05 (0.94–1.17)	0.97 (0.85–1.10)	–	0.9
	2	4096	1.0	1.07 (0.96–1.20)	0.97 (0.85–1.11)	–	0.9
Black tea	1	5707	1.0	0.95 (0.87–1.04)	0.93 (0.83–1.04)	1.00 (0.89–1.11)	0.8
	2	5682	1.0	0.96 (0.88–1.05)	0.95 (0.85–1.06)	1.02 (0.91–1.14)	0.8
Green tea^d^	1	2938	1.0	0.92 (0.83–1.03)	0.84 (0.71–0.99)	–	0.02
	2	2926	1.0	0.94 (0.84–1.06)	0.84 (0.71–1.00)	–	0.04
Herbal tea^d^	1	2206	1.0	0.98 (0.86–1.11)	0.99 (0.81–1.21)	–	0.8
	2	2201	1.0	1.03 (0.90–1.17)	1.02 (0.83–1.25)	–	0.7
Total caffeine^e^	1	5408	1.0	0.99 (0.90–1.09)	0.94 (0.85–1.04)	1.05 (0.95–1.16)	0.5
	2	5388	1.0	0.99 (0.90–1.09)	0.94 (0.85–1.04)	1.03 (0.93–1.14)	0.8

^a^Model 1: Hazard ratios (HR) and 95% confidence intervals (CI) adjusted for age and stratified by study, stage and histotype; Model 2 additionally adjusted for race, education, smoking, BMI and physical inactivity.

^b^See Table 1 for studies contributing to each model.

^c^Assessed by assigning each level a number from 0 (Q1) to 3 (Q4) and modelling this as continuous variable.

^d^The top two groups were combined ( ≥ 1 cup/day) as few individuals drank more than 2.5 cups/day.

^e^Modelled in quartiles from lowest to highest.

In stratified analyses there was no significant heterogeneity by stage of disease (Supplementary Table 1). Similarly, there was little variation by BMI, smoking status, physical inactivity, menopausal status, histotype, residual disease after surgery or study site (data not shown). The only statistically significant heterogeneity was for black tea where increasing consumption was associated with worse survival among pre-menopausal women only (HR = 1.09, 95% CI 1.01–1.17), and herbal tea where higher consumption was associated with worse survival among former smokers (HR = 1.24; 95% CI 1.07–1.44), pre-menopausal women (HR = 1.24, 95% CI 1.00–1.53) and in HAW and MCC (HR for ≥ 1 cup/day vs. none: 4.69, 95% CI 2.22–9.94 and 2.82, 1.08–7.34, respectively); however, these estimates are based on small numbers, particularly for herbal tea, so are likely due to chance.

The results were essentially the same in sensitivity analyses that included women who died in the first year and when we truncated survival at five years after diagnosis (Supplementary Table 2). They were also similar when we considered death from ovarian cancer as the outcome in the subgroup of studies that provided this information (Supplementary Table 3; HR for ≥ 1 vs. 0 cups green tea per day = 0.81, 95% CI 0.66–0.99, *p*-trend = 0.045).

## Discussion

This large international study provides support for the hypothesis that higher consumption of green tea is associated with better survival among women with ovarian cancer. We found no consistent associations between consumption of other types of tea, coffee or caffeine intake and survival overall. The minor variations we observed between sub-groups of the population are likely due to chance.

Published data regarding the relationship between tea, coffee and caffeine and ovarian cancer survival are limited. An Australian study (*N* = 609) reported no evidence of association with pre-diagnosis tea consumption but did not consider coffee or green tea [7]. In a second Australian study (*N* = 811, AUS included in this analysis), there was no significant association between higher pre-diagnosis intake of coffee, black or green tea and survival overall. However, the overall estimate for green tea was comparable to that observed here (HR for ≥ 1 vs. 0 cups/day = 0.83, 95% CI 0.60–1.15) and there was a significant dose-response with higher green tea consumption (*p* = 0.02). Excluding AUS from the current analysis did not appreciably change the magnitude of the estimate for green tea (HR for ≥ 1 vs. 0 cups per day = 0.86, 95% CI 0.68–1.08). The only study to date with information about tea consumption after diagnosis, conducted among 244 women in China, reported an inverse association between tea (predominantly green tea) and ovarian cancer-specific survival (HR = 0.43, 95% CI 0.20–0.92 for 1+ cup/day, *p*-trend <0.05) [9]. Data for other cancer types are also limited. A recent meta-analysis reported a 44% reduction in risk of recurrence for women with breast cancer (stage I or II) who drank green tea before diagnosis [21], although this was based on only two Japanese studies conducted more than 20 years ago.

The potential benefits of green tea have been attributed to the EGCG it contains. In vitro studies have shown that EGCG affects a number of signalling pathways linked to tumorigenesis, including the MAP kinase pathway which is involved in cell proliferation, differentiation and death (reviewed in [22, 23]). It also modulates inflammation and immunity, two processes that are commonly dysregulated in cancer [24]. EGCG has also been shown to inhibit tumour growth and progression in ovarian cancer cell lines [25, 26]. Although the antioxidant activity of theaflavins found in black tea is similar to that of EGCG, their total antioxidant capacity is much lower [27], potentially explaining the lack of association between black tea consumption and survival in our analysis. In contrast, a 2015 meta-analysis reported similar associations between both black tea and green tea consumption and all-cause mortality (based on 12 and 5 studies, respectively), but only black tea was associated with lower cancer-specific mortality (based on 4 and 6 studies) [28]. However, the results of that analysis are hard to interpret as the focus was on mortality not cancer survival and, of only two studies that reported all-cause mortality results for both types of tea, one reported a benefit only for black tea and the other a benefit only for green tea.

To our knowledge, this is the largest study to assess the association between tea and coffee consumption and ovarian cancer survival. We have included studies with long follow-up periods and detailed information on other covariates, including clinical and lifestyle factors. The limitations are that we only had self-reported data about pre-diagnosis consumption of tea and coffee and did not have information about the types of green tea that women drank or how it was prepared but levels of EGCG vary between brands [29] and preparation methods [30]. However, a woman’s lifestyle before diagnosis is likely to be highly correlated with her lifestyle after diagnosis and treatment [8, 31, 32]. Furthermore, any recall error or misclassification due to variation in the EGCG content of different teas is probably non-differential and so would likely have attenuated estimates for the highest vs. lowest levels of intake. Adjustment for potential confounders including level of education, smoking, BMI and physical inactivity had little impact on our estimates suggesting residual or unmeasured confounding by these factors is an unlikely explanation for the observed association with green tea. Although we cannot rule out the possibility that other factors such as variation in access to healthcare could explain the observed association, the inverse association was also seen in the Australian studies where all residents are entitled to free healthcare.

In summary, our results provide support for the hypothesis that higher consumption of green tea is associated with better survival among women with ovarian cancer. Further adequately powered studies, preferably with information about consumption after diagnosis, are needed to confirm the possible beneficial effects of green tea on ovarian cancer survival.

## Supplementary information

Supplementary Material

## Data Availability

Data cannot be made publicly available due to privacy and ethical reasons. Contact the corresponding author to discuss access to data through existing data request processes for OCAC.
