# Supplementary material for: Pre-diagnosis tea and coffee consumption and survival after a diagnosis of ovarian cancer: results from the Ovarian Cancer Association Consortium
Source: Br J Cancer. 2024 Jul 18;131(6):1043–9. doi: 10.1038/s41416-024-02792-7 (PMC11405517; doi:10.1038/s41416-024-02792-7)
Supplement: Supplementary file 1 — Supplementary Material [file 41416_2024_2792_MOESM1_ESM.pdf]

Supplementary Material

Contents

Group Members 2

AOCS Management Group: 2

OPAL Study Investigators: 2

Supplementary Table 1. Association between coffee and tea consumption and overall survival among women with early stage (local and regional) and advanced ovarian cancer 4

Supplementary Table 2. Associations between coffee and tea consumption and overall survival after a diagnosis of ovarian cancer 5

Supplementary Table 3. Associations between coffee and tea consumption and ovarian cancer survival in the subset of studies with information about cause of death (AUS, DOV, HAW, MAL, OPL) 6

Supplementary Figure 1. Forest plot showing hazard ratios (and 95% confidence intervals) for the association between consumption of ≥1 cup green tea per day (vs. none) and overall survival, by study site 7

Group Members

AOCS Management Group:

Professor David Bowtell, Peter MacCallum Cancer Centre, Melbourne, Victoria, Australia

Professor Georgia Chenevix Trench, QIMR Berghofer Medical Research Institute, Brisbane, Queensland, Australia

Professor Anna DeFazio, Department of Gynaecological Oncology, Westmead Hospital, Westmead; Centre for Cancer Research, The Westmead Institute for Medical Research & The Daffodil Centre, The University of Sydney, Sydney, New South Wales, Australia.

Professor Penelope Webb, QIMR Berghofer Medical Research Institute, Brisbane, Queensland, Australia

Ms Sian Fereday, Peter MacCallum Cancer, Melbourne, Victoria, Australia

Ms Nadia Traficante, Peter MacCallum Cancer, Melbourne, Victoria, Australia

Ms Jillian Hung, QIMR Berghofer Medical Research Institute, Brisbane, Queensland, Australia

OPAL Study Investigators:

Professor Penelope Webb, QIMR Berghofer Medical Research Institute, Brisbane, Queensland, Australia

Professor Anna DeFazio, Department of Gynaecological Oncology, Westmead Hospital, Westmead; Centre for Cancer Research, The Westmead Institute for Medical Research & The Daffodil Centre, The University of Sydney, Sydney, New South Wales, Australia.

Professor Michael Friedlander, Department of Medical Oncology, Prince of Wales Hospital, Sydney, New South Wales, Australia.

Professor Andreas Obermair, Queensland Centre for Gynaecological Cancer Research, University of Queensland, Brisbane, Queensland, Australia.

Professor Peter Grant, Gynecological Oncology Unit, Mercy Hospital for Women, Melbourne, Victoria, Australia.

Associate Professor Vanessa Beesley, QIMR Berghofer Medical Research Institute, Brisbane, Queensland, Australia

Professor Georgia Chevenix-Trench, QIMR Berghofer Medical Research Institute, Brisbane, Queensland, Australia

Professor David Bowtell Peter MacCallum Cancer Centre, Melbourne, Victoria, Australia

Associate Professor Penelope Blomfield, Royal Hobart Hospital, Hobart, Tasmania, Australia

Associate Professor Alison Brand, Department of Gynaecological Oncology, Westmead Hospital, Westmead, New South Wales and University of Sydney, Sydney, New South Wales, Australia.

Dr Alison Davis, Department of Medical Oncology, The Canberra Hospital and ANU Medical School, Australian National University, Canberra, Australian Capital Territory, Australia

Professor Yee Leung, Division of Obstetrics and Gynaecology, Faculty of Health and Medical Sciences, University of Western Australia, Crawley, Western Australia, Australia.

Associate Professor James Nicklin, Queensland Centre for Gynaecological Cancer, The Wesley Hospital, University of Qld, Brisbane, Queensland, Australia

Professor Michael Quinn, Royal Women's Hospital, Melbourne, Victoria, Australia

Ms Karen Livingstone, Consumer, Sydney, Australia

Ms Helene O'Neill, Consumer, Australia and New Zealand Gynaecology Oncology Group, Australia

Ms Merran Williams, Consumer, Queensland, Australia

Supplementary Table 1. Association between coffee and tea consumption and overall survival among women with early stage (local and regional) and advanced ovarian cancer

	None	<1 cup/day	1-2.49 cups/day	≥2.5 cups/day		
	Reference	HR (95% CI) [1]	HR (95% CI) [1]	HR (95% CI) [1]	P-trend[2]	P-het[3]
Local and regional (N)						
Coffee (2043)	1.0	0.75 (0.57-0.98)	0.83 (0.65-1.05)	0.79 (0.61-1.02)	0.2	-
Caffeinated (1471)	1.0	0.74 (0.55-0.99)	0.94 (0.73-1.22)	0.84 (0.63-1.11)	0.4	-
Decaffeinated[4] (1471)	1.0	1.02 (0.77-1.34)	1.10 (0.82-1.48)		0.6	-
Black tea (2042)	1.0	0.77 (0.63-0.95)	1.01 (0.77-1.32)	0.98 (0.73-1.31)	0.9	-
Green tea[4] (974)	1.0	0.94 (0.70-1.25)	0.77 (0.46-1.30)		0.3	-
Herbal tea[4] (704)	1.0	0.94 (0.65-1.37)	0.88 (0.44-1.79)		0.7	-
Total caffeine[5] (1950)	1.0	0.93 (0.73-1.18)	0.89 (0.70-1.15)	0.91 (0.71-1.17)	0.4	-
Distant (N)						
Coffee (3470)	1.0	0.97 (0.86-1.09)	0.99 (0.88-1.11)	1.00 (0.88-1.13)	0.9	0.2
Caffeinated (2609)	1.0	0.99 (0.87-1.13)	0.93 (0.82-1.06)	1.03 (0.91-1.17)	0.9	0.1
Decaffeinated[4] (2609)	1.0	1.09 (0.96-1.23)	0.92 (0.79-1.07)		0.7	0.2
Black tea (3466)	1.0	1.01 (0.91-1.11)	0.95 (0.84-1.08)	1.03 (0.91-1.16)	0.9	0.7
Green tea[4] (1938)	1.0	0.94 (0.83-1.06)	0.85 (0.71-1.02)		0.07	0.9
Herbal tea[4] (1404)	1.0	1.06 (0.91-1.23)	1.01 (0.81-1.26)		0.6	0.3
Total caffeine[5] (3336)	1.0	1.01 (0.91-1.13)	0.95 (0.85-1.06)	1.06 (0.95-1.18)	0.6	0.1

[1] Hazard ratios (HR) and 95% confidence intervals (CI) adjusted for age, race, education, smoking, BMI, physical inactivity; stratified by site and histotype
[2] Assessed by assigning each level a number from 0 to 2 or 3 and modeling this as continuous variable
[3] Comparison of distant vs. local/regional cancers
[4] The top two groups were combined (≥1 cup/day) as few individuals drank more than 2.5 cups/day
[5] Modelled in quartiles from lowest to highest

Supplementary Table 2. Associations between coffee and tea consumption and overall survival after a diagnosis of ovarian cancer

	N[2]	None<br>Reference	<1 cup/day<br>HR (95% CI)	1-2.49 cups/day<br>HR (95% CI)	≥2.5 cups/day<br>HR (95% CI)	P-trend[3]
Including deaths in the first year						
Coffee	5923	1.0	0.92 (0.83-1.02)	0.98 (0.89-1.08)	0.97 (0.87-1.08)	0.997
Caffeinated	4259	1.0	0.91 (0.81-1.03)	0.94 (0.84-1.05)	0.98 (0.88-1.10)	0.9
Decaffeinated[4]	4259	1.0	1.09 (0.98-1.22)	0.96 (0.84-1.10)		0.9
Black tea	5917	1.0	0.96 (0.88-1.05)	0.95 (0.85-1.06)	1.03 (0.93-1.15)	0.7
Green tea[4]	3059	1.0	0.94 (0.84-1.05)	0.84 (0.72-0.99)		0.04
Herbal tea[4]	2333	1.0	1.02 (0.89-1.16)	0.97 (0.80-1.18)		0.9
Total caffeine[5]	5594	1.0	0.99 (0.89-1.09)	0.94 (0.85-1.03)	1.03 (0.93-1.14)	0.8
Truncating survival at 5 years after diagnosis						
Coffee	5688	1.0	0.94 (0.83-1.07)	0.93 (0.82-1.04)	0.95 (0.84-1.08)	0.4
Caffeinated	4096	1.0	1.00 (0.87-1.15)	0.91 (0.79-1.04)	0.98 (0.85-1.12)	0.4
Decaffeinated[4]	4096	1.0	1.10 (0.96-1.26)	0.94 (0.80-1.11)		0.9
Black tea	5682	1.0	0.95 (0.85-1.06)	0.95 (0.83-1.08)	1.03 (0.91-1.17)	0.7
Green tea[4]	2926	1.0	0.98 (0.86-1.12)	0.85 (0.69-1.03)		0.15
Herbal tea[4]	2201	1.0	1.13 (0.97-1.32)	1.11 (0.89-1.40)		0.13
Total caffeine[5]	5388	1.0	0.97 (0.86-1.09)	0.88 (0.78-0.99)	0.99 (0.88-1.12)	0.5

[1] Hazard ratios (HR) and 95% confidence intervals (CI) adjusted for age, race, education, smoking, BMI and physical inactivity and stratified by study, stage and histotype;
[2] See Table 1 for studies contributing to each model.
[3] Assessed by assigning each level a number from 0 (Q1) to 3 (Q4) and modelling this as continuous variable
[4] The top two groups were combined (≥1 cup/day) as few individuals drank more than 2.5 cups/day
[5] Modelled in quartiles from lowest to highest

Supplementary Table 3. Associations between coffee and tea consumption and ovarian cancer survival in the subset of studies with information about cause of death (AUS, DOV, HAW, MAL, OPL)

		None	<1 cup/day	1-2.49 cups/day	≥2.5 cups/day	
	N[2]	Reference	HR (95% CI)[1]	HR (95% CI)[1]	HR (95% CI)[1]	P-trend[3]
Coffee	2997	1.0	0.89 (0.76-1.04)	0.95 (0.82-1.10)	0.94 (0.80-1.09)	0.7
Caffeinated	1929	1.0	0.88 (0.73-1.06)	0.89 (0.73-1.08)	0.93 (0.78-1.11)	0.6
Decaffeinated[4]	1929	1.0	1.21 (1.00-1.47)	0.92 (0.69-1.22)	-	0.6
Black tea	2999	1.0	0.99 (0.86-1.14)	0.98 (0.83-1.16)	1.12 (0.97-1.31)	0.1
Green tea[4]	1922	1.0	0.94 (0.81-1.09)	0.81 (0.66-0.99)	-	0.045
Herbal tea[4]	1925	1.0	1.06 (0.91-1.23)	0.94 (0.75-1.18)	-	0.98
Total caffeine[5]	2934	1.0	1.02 (0.88-1.18)	0.99 (0.86-1.15)	0.98 (0.84-1.13)	0.7

[1] Hazard ratios (HR) and 95% confidence intervals (CI) adjusted for age, race, education, smoking, BMI and physical inactivity and stratified by study, stage and histotype;
[2] See Table 1 for specific studies contributing to each model.
[3] Assessed by assigning each level a number from 0 (Q1) to 3 (Q4) and modelling this as continuous variable
[4] The top two groups were combined (≥1 cup/day) as few individuals drank more than 2.5 cups/day
[5] Modelled in quartiles from lowest to highest

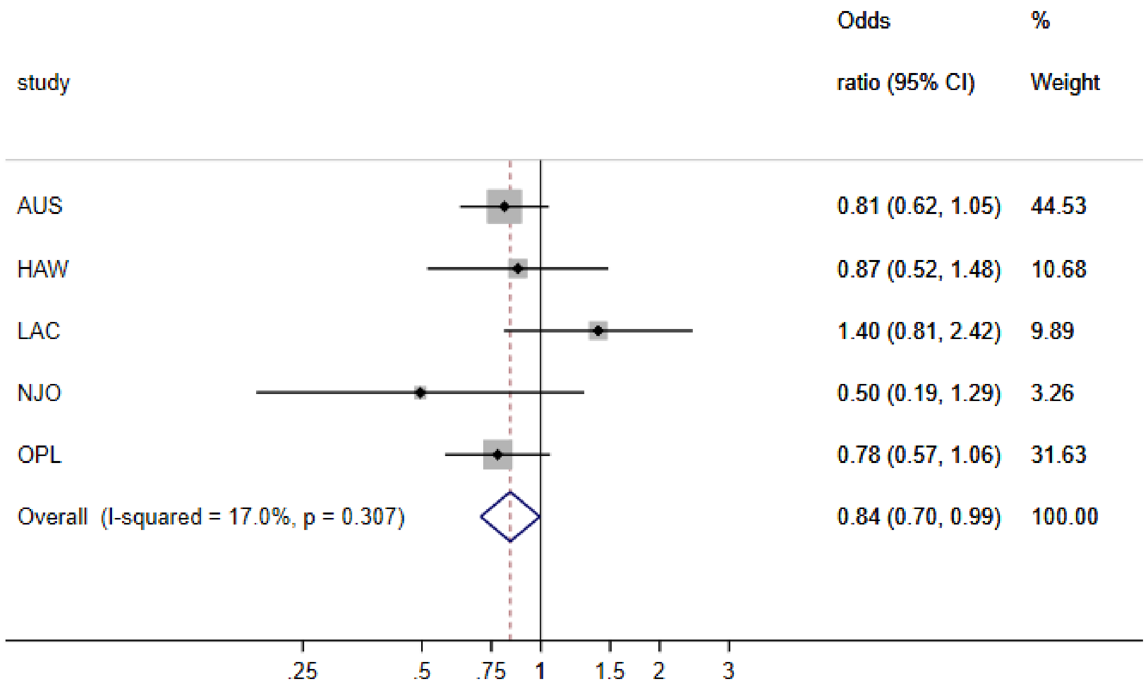

Supplementary Figure 1. Forest plot showing hazard ratios (and 95% confidence intervals) for the association between consumption of ≥1 cup green tea per day (vs. none) and overall survival, by study site
